# Association between thymic hyperplasia and serum calcium level in Graves’ disease

**DOI:** 10.1186/s12902-024-01541-4

**Published:** 2024-01-29

**Authors:** Jing Zeng, Lan Li, Dong Wei

**Affiliations:** 1 Department of Endocrinology and Metabolism, The Second People’s Hospital of Chengdu, No.10 Qingyunan Street, Jinjiang District, Chengdu, 610017 China; 2 Medical Examination Center, The Second People’s Hospital of Chengdu, No.10 Qingyunan Street, Jinjiang District, Chengdu, 610017 China; 3 Department of General Internal Medicine, West China Second University Hospital, Sichuan University, No.20, Section 3, Ren Min Nan Lu, 610041 Chengdu, China

**Keywords:** Graves’ disease, Thymic hyperplasia, Calcium, Bone metabolism

## Abstract

**Background:**

Graves' disease increases bone resorption in hyperthyroidism, leading to elevated serum calcium levels and a negative bone balance. Thymic hyperplasia is observed in some Graves' disease patients. What's more, there have been a few reports of increased serum calcium and severe osteoporosis induced by Graves’ disease with thymic hyperplasia. It remains unclear whether Graves’ disease with thymic hyperplasia is associated with higher serum calcium levels. Our study aimed to investigate the possibility of elevated serum calcium levels and aggravated bone mobilization in Graves’ disease patients with thymic hyperplasia.

**Methods:**

Newly diagnosed and untreated patients with Graves' disease (*n* = 96) were enrolled. They were divided into two groups based on the incidental detection of thymic hyperplasia during imaging. Albumin, alkaline phosphatase, calcium, free triiodothyronine, free thyroxine, thyroid-stimulating hormone, and thyrotrophin receptor antibody (TRAb) were measured, and a computerized tomography of the chest was obtained.

**Results:**

Patients with Graves’ disease who had thymic hyperplasia were notably younger (*P*=0.018) and exhibited higher serum calcium levels (*P*=0.001) compared to those with Graves’ disease without thymic hyperplasia. In the multiple regression analysis, thymic hyperplasia, TRAb, and female gender were significant variables associated with elevated serum calcium levels in patients with Graves' disease, collectively accounting for 31.7% of the variation in serum calcium.

**Conclusions:**

Graves’ disease patients with thymic hyperplasia showed higher serum calcium levels. thymic hyperplasia, TRAb, and female gender were found to be correlated with increased serum calcium levels in Graves’ disease, suggesting a potential association between thymic hyperplasia and bone mobilization in Graves’ disease.

## Background

The global prevalence of hyperthyroidism is approximately 0.2% to 1.3% in iodine-abundant areas [1]. Graves’ disease (GD) is the most common cause of hyperthyroidism, accounting for 70%–80% in iodine-sufficient areas and 50% in iodine insufficient areas, respectively [1, 2]. GD is an autoimmune disease that leads to the development of antibodies against the thyrotropin receptor, resulting in a clinical state of thyrotoxicosis. Thyrotoxicosis promotes bone resorption more than bone formation [3], ultimately leading to a negative bone balance and elevated serum calcium levels [4]. Hypercalcemia occurs in about 15% to 20% of hyperthyroidism patients [5], but the increase in serum calcium due to hyperthyroidism is typically mild and associated with only slight symptoms.

In clinical practice, thymic hyperplasia is often incidentally diagnosed during chest imaging conducted for unrelated reasons. However, the presence of thymic hyperplasia is not coincidental; many case reports and series have described this association [6–10]. Studies by Michi et al. have indicated that approximately 38% of thyrotoxicosis patients have thymic hyperplasia. Nonetheless, the real incidence of thymic hyperplasia in GD remains unknown. Furthermore, a subset of GD patients with thymic hyperplasia shows higher serum calcium levels and more severe osteoporosis [11–13]. It is unclear whether Graves' disease with thymic hyperplasia is indicated by higher serum calcium levels compared to Graves' disease alone. The primary aim of our investigation is to evaluate elevated serum calcium levels and the possibility of aggravating bone mobilization in GD patients with thymic hyperplasia.

## Methods

### Subjects

Three hundred and fifty-five adult patients with Graves’ disease were evaluated in our inpatient department between January 2018 and June 2020. Ninety-six patients with Graves’ disease who were newly diagnosed and untreated were enrolled. The patients were divided into two groups according to their thymic status in the study (thymic hyperplasia *n* = 19 and no-thymic hyperplasia *n* = 77).

The inclusion criteria were as follows: meeting the diagnostic criteria of Graves' disease and being over 18 years of age. The exclusion criteria included a personal history of fracture prior to the onset of the disease, non-thyroidal illness (such as parathyroid diseases, liver disease, renal dysfunction, malignancy, diabetes mellitus, hypercortisolism, or hypogonadism); intake of drugs (bisphosphonates, calcitonin, testosterone, steroids, diuretics, heparin, lithium, or anticonvulsants); and pregnancy that could affect bone metabolism. All subjects underwent chest computed tomography, and patients with thymic lesion showing a malignant tendency were excluded. This study was approved by the Research Ethics Committee of Chengdu Second People’s Hospital (No. 2021020). The written informed consent requirement was waived by the Ethics Committee at Chengdu Second People's Hospital due to the retrospective nature of the study and data collection from routine clinical practice.

The diagnosis of Graves’ disease had been established based on the presence of symptoms and signs of hyperthyroidism, a diffuse goiter, ophthalmopathy, and/or positive thyrotrophin receptor antibody, suppressed thyroid-stimulating hormone, and high or normal serum concentrations of free thyroxine and free triiodothyronine.

We measured simultaneous PTH levels when serum calcium exceeded the upper limit of normal and excluded patients with abnormal PTH levels when serum calcium returned to normal after fluid replacement and/or propranolol treatment (without antithyroid drugs). This initial step aimed to rule out the possibility that hypercalcemia might be influenced by hyperparathyroidism or parathyroid hormone-related peptide (PTHrP).

### Biochemical measurements

Blood samples were collected after an overnight fast. Serum concentrations of albumin (Alb), alkaline phosphatase (ALP), and calcium (Ca) were measured using the HITACHI LABOSPECT 008 automatic analyzer at our hospital's central laboratory. Serum calcium levels were corrected when serum albumin was not at 40 g/L. Free triiodothyronine (FT3), free thyroxine (FT4), and thyroid-stimulating hormone (TSH) were assessed using the Abbott i4000 SR chemiluminescence instrument (chemiluminescent microparticle immunoassay; interassay coefficient of variation (CV) ≤10%). Thyrotrophin receptor antibodies (TRAb) were measured with the Roche COBAS 6000 e601 (electrochemical luminescence assay, interassay CV ≤10%). The normal reference ranges for the variables were as follows: TSH 0.35-4.95 μIU/mL, FT4 9.00-19.00 pmol/L, FT3 2.67-5.70 pmol/L, ALP 45-125 U/L, TRAb <1.75 IU/L, Ca 2.11-2.52 mmol/L.

### Statistical analysis

Results are expressed as mean ±SD for normally distributed data, as percentage when data passed the Kolmogorov-Smirnov normality test, or as median and range for non-normally distributed data. Group differences were analyzed using the Student’s T-test for normally distributed data and the Mann–Whitney’s U test for non-parametric data. Correlations were analyzed using the Spearman correlation coefficient. Multiple linear regressions were performed to identify the factors influencing serum calcium levels. A *p*-value <0.05 was considered statistically significant. Statistical analysis was conducted using SPSS software (version 25.0, IBM Corporation).

## Results

**Fig. 1:**
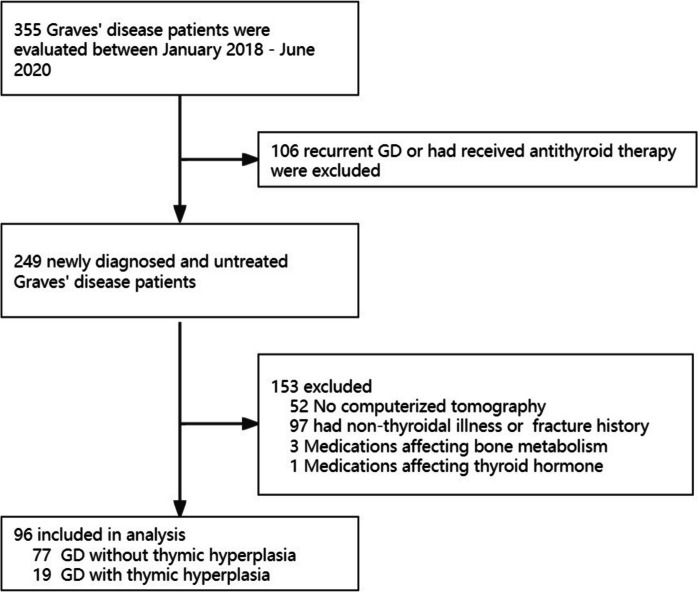
Flow diagram of participants selection

Figure 1 shows the flow chart of subject enrollment for Graves’ disease. A total of 355 patients with GD were assessed during the study period. 106 patients were excluded due to recurrent GD or prior antithyroid therapy. An additional 52 patients without computed tomography scans were excluded. 97 patients with previous fractures or non-thyroidal illnesses mentioned above were also excluded. 4 patients taking medications that could affect thyroid hormone levels or bone metabolism were excluded. The baseline characteristics of the GD patients are presented in Table 1.

**Table 1:** Baseline characteristics of patients with Graves’ disease

Parameter	thymic hyperplasia	No-thymic hyperplasia	*P* Value
	(*n* = 19)	(*n* = 77)	
Age(year)	42.6±11.3	51.6±15.0	0.018
Gender			0.564
Male	7(36.8%)	34(44.2%)	
Female	12(63.2%)	43(55.8%)	
Ca (mmol/L)	2.40±0.13	2.30±0.11	0.001
ALP (IU/L)	108.78±36.50	92.05±36.30	0.076
TRAb(IU/L)	10.23 (3.99-18.74)	7.70 (3.39-18.80)	0.499
TSH(mIU/L)	0.000 (0.000-0.001)	0.000 (0.000-0.001)	0.689
FT3(pmol/L)	18.66 (9.58-47.00)	16.21 (9.73-22.91)	0.492
FT4(pmol/L)	28.88 (20.12-40.02)	29.58 (24.90-40.32)	0.547

*Ca* Calcium, *ALP* Alkaline phosphatase, *FT3* Free triiodothyronine, *FT4* Free thyroxine, *TSH* Thyrotropin, *TRAb* Thyrotrophin receptor antibodies

**Fig. 2:**
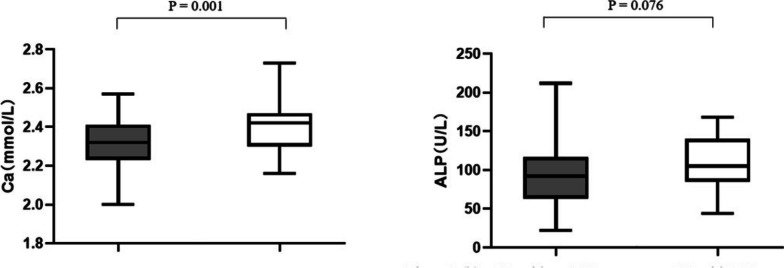
Boxplots showing serum levels of calcium (**a**) and alkaline phosphatase (**b**) in our study while patients were either GD with Thymic hyperplasia or GD without Thymic hyperplasia. The box represents the interquartile range with the thicker black line being the median. TH, Thymic hyperplasia; Ca, calcium; ALP, alkaline phosphatase

They were categorized into two groups based on the presence or absence of incidentally detected thymic hyperplasia in imaging. The group with thymic hyperplasia was significantly younger (*P* = 0.018) and had higher serum calcium levels (*P* = 0.001) than the group without thymic hyperplasia. Although the level of ALP in GD patients with thymic hyperplasia was higher than that in GD patients without thymic hyperplasia, the difference was not statistically significant (*P* = 0.076) (Fig. 2). We compared TSH, FT4, FT3, and TRAb titers and found no significant difference between the two groups.

TRAb, gender, age, thymus hyperplasia, ALP, FT4 and FT3 were included in the Spearman correlation analysis. The results indicated a positive correlation between serum calcium levels and gender, TRAb, FT4, FT3 and thymus hyperplasia (*P* < 0.05) (Table 2).

**Table 2:** Correlations between Ca, TRAb, FT4, FT3, thymic hyperplasia and gender

		TRAb	Thymic hyperplasia	Female	FT4	FT3
Ca	r	0.452	0.294	0.263	0.212	0.406
	P	0.000	0.014	0.019	0.038	0.000

*Ca* Calcium, *TRAb* Thyrotrophin receptor antibodies, *FT4* Free thyroxine, *FT3* Free triiodothyronine

In the multiple-line regression, we used serum calcium as the dependent variable and TRAb, FT4, FT3, gender, age and thymus status as independent variables, according to the correlation analysis and the conclusions of previous literature research. The results showed that thymic hyperplasia (β = 0.087, *P* = 0.036), TRAb (β = 0.003, *P* = 0.006), and female (β = 0.051, *P* = 0.028) were significant variables in relation to elevated serum calcium in patients with Graves' disease, accounting for 31.7% of the variation in serum calcium (*R*^2^ = 0.317) (Table 3).

**Table 3:** The results of multiple liner regression analysis

	*β coefficient*	*95% CI*	*P value*
age	-0.009	-0.002 to 0.002	0.926
Female	0.051	0.006 to 0.097	0.028
Thymic hyperplasia	0.087	0.029 to 0.144	0.035
TRAb	0.003	0.001 to 0.005	0.006
FT3	0.000	0.000 to 0.001	0.330
FT4	0.001	-0.001 to 0.003	0.179

*FT3* Free triiodothyronine, *FT4* Free thyroxine, *TRAb* Thyrotrophin receptor antibodies

## Discussion

Hyperthyroidism is a well-established factor in the development of hypercalcemia. It stimulates the differentiation of osteoblasts and osteoclasts, resulting in an acceleration of both bone resorption and bone formation processes [14]. Since bone resorption increased to a greater extent than bone formation [3], the ultimate result is a negative bone balance [4] and hypercalcemia [15]. However, in cases of hypercalcemia induced by thyrotoxicosis alone, serum calcium levels rarely exceed 3mmol/L and are usually asymptomatic [3]. Halsted first reported thymic hyperplasia in patients with Graves' disease (GD) in 1914 [16]. The mechanisms underlying thymic changes in GD patients have not been fully elucidated. It is widely accepted that thymic hyperplasia is an effect of the autoimmune processes associated with Graves’ disease rather than a cause of the disease itself [12]. The majority of thymic lesions in GD are benign. Furthermore, Studies by Murakami et al. [17] and Yacoub et al. [18] have shown that antithyroid drug treatment significantly reduces thymic enlargement and density. Therefore, it is generally recommended to closely monitor the thymus during the treatment of hyperthyroidism rather than performing active thymus biopsy and thyroidectomy, unless imaging indicates a potential malignancy. Previous case reports have suggested that hyperthyroidism combined with thymic hyperplasia may lead to a more significant elevation of serum calcium and even osteoporosis [11–13]. However, there has been no prior investigation into the correlation between them. To the best of our knowledge, our study is the first to investigate the association between thymic hyperplasia and serum calcium levels and bone metabolism in GD patients.

In our present study, the results demonstrated a significant disparity in serum calcium levels between GD patients with and without thymic hyperplasia. Although the level of alkaline phosphatase (ALP) in GD patients with thymic hyperplasia was higher, this difference did not reach statistical significance. From the design of the trial, we rule out the possibility that this difference in calcium levels is due to differences in sex, long disease duration, or treatment regimen between the two groups. Furthermore, there were no variations in thyroid function observed between the two GD groups, indicating that the differences were not linked to the severity of thyrotoxicosis.

In the multiple regression analysis, thymic hyperplasia, gender (female) and thyrotrophin receptor antibody (TRAb) titer emerged as significant variables associated with elevated serum calcium. The mechanism by which thymic hyperplasia influences the increase in serum calcium levels remain unclear. However, pathological studies suggest that thymic hyperplasia in GD patients is driven by immune and thyroid hormone-dependent pathogenesis. We hypothesize that the elevated serum calcium levels in GD patients with thymic hyperplasia are also influenced by these underlying mechanisms.

First, previous research by Kim et al. and Dutton et al. has reported thymic tissue can express thyrotropin receptor (TSHR) [19, 20]. Additionally, Haider et al. [9] proposed a positive correlation between the degree of thymic growth and TRAb titer. Young et al. [21] and Geanina et al. [10] have observed dynamic changes in thymic hyperplasia through serial computed tomography images that coincide with variations in serum TSHR antibody levels, with a shrinking of the thymus as TRAb titers decrease. Simultaneously, previous studies have confirmed that TRAb plays a very important role in bone metabolism. These studies, conducted on both males and pre- or postmenopausal women, have consistently found a negative correlation between TRAb titers and bone mineral density [22–24]. Moreover, Kumeda et al. [25] have reported a close association between TRAb and bone metabolic markers in Graves' disease. In contrast, others [26–28] published reports that TRAb may have a bone-preserving effect through its interaction with TSH receptors. The correlation between TRAb titers and their effects on bone, as found in the present study and in some other studies, is not well explained. One possible reason for this discrepancy may be the use of TRAb instead of thyroid-stimulating antibody (TSAb) in previous studies. It is worth noting that certain investigations have demonstrated a significantly negative correlation between serum TSAb titers and lumbar vertebral bone mineral density, as well as a positively correlation with bone metabolism markers [29, 30]. Diana et al. observed that TSAb dose-dependently increased superoxide release in human embryonic kidney-293 cells. Their research revealed that oxidative stress markers in GD patients exceeded those of patients with toxic nodular goiter or healthy euthyroid subjects [15]. According to these findings, GD with thymic hyperplasia may exacerbate bone mobilization by triggering a more pronounced immunological response.

Nvertheless, Jinguji et al. [7] reported a significant reduced in thymic size and density following I-131 therapy for Graves’ disease, despite concurrent increase in serum TRAb levels. This finding suggests a potential association between thymic hyperplasia, the exacerbation of thyrotoxicosis, and the promotion of bone mobilization. The hyperthyroid state is known to induce hyperplasia of the thymic cortex and medulla intima, resulting in thymic growth [9, 31]. Early animal experiments also noted thymic enlargement in thyroxine-treated rats [32], which subsequently regressed after thyroidectomy [17]. Moreover, clinical observations have indicated a reduction in thymus size during the course of hyperthyroidism treatment in and remission. Simpson et al. [33] and Scheiff et al. [34] demonstrated the impact of thyroid hormones on thymic epithelial cells. Furthermore, Scheiff et al. noted that thyroid hormones had no discernible effect on medullary lymphocytes, despite the proliferation of cortical lymphocytes. Numerous previous studies have established a correlation between thymic volume and thyroid hormone levels in patients undergoing antithyroid therapy [6, 18]. Regrettably, we did not observe differences in thyroid function in our study, which may be attributed to our study population primarily consisting of untreated Graves' disease patients.

Nevertheless, it is somewhat unexpected that the effect of thymic hyperplasia on serum calcium was independent of TRAb and thyroid hormones in our multiple regression analysis. The underlying pathophysiological mechanism is still unclear. Giovanella et al. reported elevated levels of parathyroid hormone-related peptide in patients with Graves' disease and thymic hyperplasia [11], which could lead to or aggravate the elevation of serum calcium. With the remission of hyperthyroidism and the reduction of the thymus size, parathyroid hormone-related peptide gradually returned to normal levels. In summary, we propose that higher TRAb titers and/or more severe thyrotoxicosis increase the likelihood of thymic hyperplasia, subsequently contributing to bone mobilization. However, other potential mechanisms still need to be elaborated by further research.

It is well-established that serum calcium levels decrease with advancing age, particularly in females. However, in untreated patients with hyperthyroidism, as age increases, especially in postmenopausal women, the higher susceptibility to bone resorption and osteoporosis, due to the absence of estrogen's protective effects, might lead to elevated blood calcium levels. Intriguingly, in our study, younger patients with thymic hyperplasia presented with higher serum calcium levels, suggesting a potential role of thymic hyperplasia in increasing serum calcium levels in hyperthyroidism. In a similar manner, our study indicated that GD patients with thymic hyperplasia tended to be younger, which is consistent with prior research [8, 10]. The precise reasons for this age difference remain unclear and warrant further investigation for clarification.

The limitations of our study include potential biases in patient selection attributable to the retrospective nature of the study. A relatively small study population represents another major limitation. Given the retrospective design, we could not gather comprehensive markers of bone metabolism, 25-OH-vitamin D, calcitriol, and dietary calcium intake for the study cohort. Additionally, PTH was only assessed in patients with hypercalcemia. Owing to resource constraints, the detection of PTHrP was not feasible. Performing 99mTc-MIBI scintigraphy in the future studies would enhance the accuracy of ruling out PHPT. These unmeasured confounding facorts should be taken into account in future studies.

## Conclusions

In conclusion, GD patients with thymic hyperplasia exhibited higher serum calcium levels. Thymic hyperplasia, female gender, and TRAb titers were correlated with increased serum calcium levels in GD, indicating their association with bone metabolism in the condition. For patients with concurrent thymic hyperplasia, active treatment is advisable to mitigate the risk of hypercalcemia and loss of bone mineral density.

## Data Availability

The datasets generated during this study are available from the correspondences on reasonable request.

## Abbreviations

TRAb: Thyrotrophin receptor antibody
GD: Graves’ disease
Alb: Serum concentrations of albumin
ALP: Alkaline phosphatase
Ca: Calcium
FT3: Free triiodothyronine
FT4: Free thyroxine
TSH: Thyroid-stimulating hormone
TSHR: Thyrotropin receptor
TSAb: Thyroid-stimulating antibody
